# Clinical and Ultrasound Thyroid Nodule Characteristics and Their Association with Cytological and Histopathological Outcomes: A Retrospective Multicenter Study in High-Resolution Thyroid Nodule Clinics

**DOI:** 10.3390/jcm8122172

**Published:** 2019-12-09

**Authors:** María Molina-Vega, Carlos Antonio Rodríguez-Pérez, Ana Isabel Álvarez-Mancha, Gloria Baena-Nieto, María Riestra, Victoria Alcázar, Ana Reyes Romero-Lluch, Juan C. Galofré, José C. Fernández-García

**Affiliations:** 1 Department of Endocrinology and Nutrition, Instituto de Investigación Biomédica de Málaga (IBIMA), Virgen de la Victoria University Hospital, 29010 Málaga, Spain; 2 Department of Endocrinology, Insular University Hospital of Gran Canaria, 35016 Las Palmas de Gran Canaria, Spain; carlosrod81@hotmail.es; 3 Department of Pathology, Virgen de la Victoria University Hospital, 29010 Málaga, Spain; 4 Department of Endocrinology, Jerez Hospital, 11408 Jerez de la Frontera, Cádiz, Spain; gloria.baenanieto@gmail.com; 5 Department of Endocrinology, University Hospital of Cabueñes, 33394 Gijón, Spain; mriestra.fernandez@gmail.com; 6 Department of Endocrinology, Hospital Severo Ochoa, 28911 Leganés, Madrid, Spain; victoria.alcazar@gmail.com; 7 Department of Endocrinology, Hospital Universitario Virgen del Rocío, 41013 Sevilla, Spain; anaromerolluch@hotmail.com; 8 Department of Endocrinology, Clínica Universidad de Navarra, 31008 Pamplona, Spain; jcgalofre@unav.es

**Keywords:** thyroid nodules, ultrasound, fine needle aspiration

## Abstract

Introduction: Thyroid nodules are a common finding. A high-resolution thyroid nodule clinic (HR-TNC) condenses all tests required for the evaluation of thyroid nodules into a single appointment. We aimed to evaluate the clinical outcomes at HR-TNCs. Design and Methods: A retrospective cross-sectional multicenter study including data from four HR-TNCs in Spain. We evaluated fine-needle aspiration (FNA) indications and the association between clinical and ultrasound characteristics with cytological and histopathological outcomes. Results: A total of 2809 thyroid nodules were included; FNA was performed in 82.1%. Thyroid nodules that underwent FNA were more likely larger, isoechoic, with microcalcifications, and in younger subjects. The rate of nondiagnostic FNA was 4.3%. A solid component, irregular margins or microcalcifications, significantly increased the odds of Bethesda IV-V-VI (vs. Bethesda II). Irregular margins and a solid component were independently associated with increased odds of malignancy. Thyroid nodules <20 mm and ≥20–<40 mm had a 6.5-fold and 3.3-fold increased risk for malignancy respectively in comparison with those ≥40 mm. Conclusion: In this large multicenter study, we found that the presence of a solid component and irregular margins are factors independently related to malignancy in thyroid nodules. Since nodule size ≥40 mm was associated with the lowest odds of malignancy, this cut-off should not be a factor leading to indicate thyroid surgery. HR-TNCs were associated with a low rate of nondiagnostic FNA.

## 1. Introduction

Thyroid nodules, described as discrete lesions within the thyroid gland that are radiologically distinct from the surrounding thyroid parenchyma [1], are a common clinical finding. Their prevalence has increased in parallel with the widespread use of imaging tests, mainly neck ultrasound (US), and the increasing awareness of health care professionals. Overall, thyroid nodules are found by palpation in 4%–8% of adults, disclosed by US examination in 20%–67% of patients, and found in 50% of necropsies [2].

When assessing a thyroid nodule, the primary objective is to differentiate a benign thyroid nodule from that harboring thyroid cancer. Although most thyroid nodules are benign lesions [3], approximately 7%–15% of them may represent thyroid cancer [1].

Fine-needle aspiration (FNA), given its high sensitivity and specificity [4], is the test of choice for the detection of malignant nodules [5]. Performing an FNA, however, is not always necessary, and to stratify the risk of malignancy and the indication for FNA, different thyroid nodule US classification systems have been developed [1,5,6,7].

Suspicious sonographic signs in a thyroid nodule include the presence of microcalcifications, irregular margins, hypoechoic parenchyma in comparison with the surrounding thyroid/strap muscles and being taller-than-wide. In addition, a cystic component of the thyroid nodule (in absence of suspicious US data), in comparison with a solid component, is considered a favorable US feature [1]. Importantly, although FNA-recommended cut-off sizes differ across the classification systems, thyroid nodules with a diameter <1 cm usually do not need to undergo FNA, unless they present with more than one suspicious US characteristic. Similarly, in those nodules described as partially cystic or spongiform, FNA should be considered only if the maximum diameter is more than 2 cm [1,8].

On the other hand, in some centers, the diagnostic workup of a thyroid nodule involves a significant number of medical visits, a long delay, and a significant inconvenience for patients (loss of working hours, economic impact, and anxiety due to diagnosis delay). The implementation of high-resolution thyroid nodule clinics (HR-TNC) provide a one-stop thyroid nodule evaluation that includes focused medical assessment, US examination, and FNA, all performed within a 60 min appointment [9].

In this multicenter study involving HR-TNCs, we aimed to analyze the US and clinical characteristics of thyroid nodules leading to the indication of FNA, and we evaluated the association of these characteristics with cytological and histopathological outcomes.

## 2. Experimental Section

### 2.1. Study Population

This was a retrospective cross-sectional study including data from 2013 to 2016 from four different HR-TNCs in Spain (Virgen de la Victoria University Hospital, Malaga; Insular University Hospital of Gran Canaria, Las Palmas de Gran Canaria; University Hospital of Jerez, Jerez de la Frontera, Cádiz; and University Hospital of Cabueñes, Gijón).

The inclusion criteria for the present study were data availability regarding thyroid nodule size (the largest diameter of the thyroid nodule) and the following sonographic characteristics: echogenicity (hyperechoic, isoechoic or hypoechoic), composition (solid or mixed), margins (regular or irregular) and microcalcifications (absent or present). Only solitary thyroid nodules were included. Simple thyroid cysts were excluded.

US examination of the thyroid was performed by a radiologist or an endocrinologist with expertise in thyroid sonography, using a 10 to 18 MHz transducer. The largest diameter of the nodule, echogenicity, parenchyma composition, margin characteristics and presence of microcalcifications were evaluated. In addition, a serum thyrotropin (TSH) level was obtained in all patients. If serum TSH was subnormal, a thyroid scintigraphy was performed and thyroid nodules were classified as hyperfunctioning, isofunctioning or nonfunctioning. Hyperfunctioning nodules did not undergo FNA as they rarely harbor malignancy [1].

FNA was performed according to the American Thyroid Association (ATA) guidelines under US guidance using a 25 gauge needle. Surgery was proposed according to US and FNA results and the clinical situation of patients. Both aspiration and surgical specimens were evaluated by a cytopathologist, and thyroid cytopathology was categorized using the 2009 Bethesda System [10].

### 2.2. Study Outcomes

We evaluated three main outcomes in our study: (1) clinical and sonographic characteristics associated with FNA, (2) clinical and sonographic characteristics leading to surgery referral (Bethesda II vs. Bethesda IV-V-VI), and (3) clinical and sonographic characteristics associated with histological malignancy.

### 2.3. Ethics

This study was reviewed and approved by the Ethics Committee of each participating center and was conducted according to the principles of the Declaration of Helsinki. The participants provided signed consent after being fully informed of the study goal and its characteristics.

### 2.4. Statistical Analysis

All statistical analyses were performed using SPSS Statistics (version 25 for Windows; IBM Corp., Armonk, NY, USA). For statistical analysis, thyroid nodules were divided into three groups based on maximum diameter: <20 mm, ≥20–<40 mm and ≥40 mm. Data were expressed as the mean ± SD for quantitative variables and percentages for categorical variables Comparisons between the study groups of the variables were performed using Student’s *t* test. Group comparisons of qualitative data were performed using the chi-square test.

Univariate logistic regression was used to estimate the odds ratios (OR) of performing FNA, presenting Bethesda IV-V-VI in the cytopathological analysis and being a malignant nodule. Next, a parsimonious multivariate logistic regression model was constructed, taking into account multicollinearity (through the variance inflation factor). The criterion used for selecting the best model was based on the Akaike information criterion. Significance for all statistical tests was set at *p* < 0.05 for two-tailed tests.

## 3. Results

### 3.1. General Characteristics

A total of 2809 subjects with solitary thyroid nodules were identified. Table 1 shows their clinical, US and cytopathological characteristics. Briefly, the mean age of the participants was 51.9 ± 14.3 years, and 82.7% of nodules appeared in women. Regarding thyroid function, 83.9%, 11.8% and 4.2% of patients had normal levels of thyroid hormones, hypothyroidism and hyperthyroidism, respectively. The mean diameter was 21.8 ± 1 2.3 mm, and most nodules were hypoechoic (58%) and solid (70.4%). The prevalence of irregular margins and microcalcifications was below 4% and 7.3%, respectively.

Based on US characteristics, size, or prior clinical history, 2308 nodules (82.1%) underwent FNA. In the cytopathological study, 81.2% were classified as benign, 11.4% were indeterminate (6.1% Bethesda III, 5.3% Bethesda IV), 0.9% were suspected to have malignancy, 2.2% were malignant, and 4.3% were inadequate for diagnosis (Table 1).

### 3.2. Surgical Treatment

Among all FNA-analyzed nodules, surgery was performed in 25.3% (585 nodules)—of which, 1.2% were Bethesda I, 50.4% were Bethesda II, 18.1% were Bethesda III, 18.8% were Bethesda IV, 3.1% were Bethesda V and 8.4% were Bethesda VI. Total thyroidectomy was performed in 67.9% of cases, and hemithyroidectomy was performed in 32.1%. Regarding the reasons for surgery, in the Bethesda II category, surgery was recommended due to nodule size, patient preference and hyperthyroidism in 94.8%, 4% and 1% of cases, respectively. In the Bethesda IV category, 93.5% of patients underwent surgery because of the cytopathological results, and in 6.5%, surgery was also indicated due to the thyroid nodule size. In all Bethesda V and Bethesda VI cases, surgery was recommended due to the cytopathological results. Most Bethesda I thyroid nodules were referred to surgery due to size (76.9%), whereas Bethesda III thyroid nodules were referred to surgery mostly due to the cytopathological results (88.3%).

The final diagnoses, based on histopathological analysis of the surgical specimen, were as follows: 79.8% benign, 17.2% papillary carcinoma, 1.3% follicular carcinoma, 0.5% medullary carcinoma and 1.2% Hürthle cell carcinoma. The diagnostic concordance between cytopathological and histopathological examinations was as follows: Bethesda II, 97% benign; Bethesda III, 81% benign; Bethesda IV, 69.9% benign; Bethesda V, 70.6% malignant and Bethesda VI, 90% malignant.

### 3.3. Association between Thyroid Nodule Characteristics and FNA Performance, Cyto- and Histopathological Outcomes

Nodules that underwent FNA were from younger patients, larger, more frequently palpable, isoechoic and mixed, and presented microcalcifications in a higher proportion than those not biopsied (Table 2). In the multivariate logistic regression analysis, we found that a larger nodule size, isoechogenicity and the presence of microcalcifications significantly increased the odds of performing FNA (Table 3).

On the other hand, nodules in the Bethesda IV-V-VI group presented with a higher proportion of solid components, irregular margins and microcalcifications than nodules from the Bethesda II group (Table 2). In the multivariate logistic regression analysis (Table 4), the presence of irregular margins was the most important determinant for a Bethesda IV-V-VI diagnosis (2.6-fold increase), followed by presenting with a solid component and the presence of microcalcifications. Nodule size, however, was not related to the odds of a Bethesda IV-VI-VI diagnosis in the multivariate analysis.

Of 585 nodules that were subjected to surgery, 23.1% were malignant. Malignant nodules were significantly smaller, more hypoechoic, and more frequently presented with irregular margins and microcalcifications than the benign nodules (Table 2). The malignancy rate was 39.7% for nodules <20 mm, 20.3% for nodules ≥20–<40 mm and 6.6% for those ≥40 mm (*p* < 0.001). Last, in the multivariate logistic regression analysis, thyroid nodules with irregular margins presented a 5.6-fold higher risk for malignancy than thyroid nodules with regular margins, and a solid component more than doubled the risk of malignancy with respect to the risk associated with mixed cystic and solid nodules. Of note, in this multivariate analysis, thyroid nodules ≥40 mm presented the lowest odds of malignancy, while nodules <20 mm and ≥20–<40 mm had a 6.5- and 3.3-fold increased risk, respectively, of being malignant in comparison with thyroid nodules ≥40 mm (Table 5).

## 4. Discussion

In this multicenter study involving four HR-TNCs in Spain, we evaluated the US and clinical characteristics of thyroid nodules leading to the indication for FNA and analyzed the association of these characteristics with cytological and histopathological outcomes. Our main findings indicate that in this HR-TNC setting, a younger age, larger thyroid nodule size, the presence of microcalcifications and an isoechogenic US pattern are factors associated with higher odds of undergoing FNA. Additionally, we found that nodules with a solid component presenting irregular margins and microcalcifications have a higher risk of being suspicious/malignant (Bethesda IV-V-VI), whereas a solid component, irregular margins and a size <40 mm were the factors most related to thyroid nodule malignancy.

Consistent with previous data from the literature [11], the vast majority of patients with thyroid nodules were women. Although male sex has been described as a risk factor for thyroid nodule malignancy [12], we did not observe any sex difference in our study. Regarding age, although the prevalence of clinically relevant thyroid nodules increases with advancing age, the risk that such nodules are malignant decreases [13,14]. Thus, in our population, FNA was more likely to be indicated in younger subjects, but we did not observe any relationship between increasing age and malignancy rate.

Interestingly, although a larger nodule size was clearly related to a higher likelihood of undergoing FNA, there was an inverse relationship between nodule size and malignancy, with thyroid nodules ≥40 mm being associated with the lowest risk for malignancy, while those <20 mm and ≥20–<40 mm had more than a 6-fold and 3-fold increased risk for malignancy, respectively. We attribute these findings to the fact that many Bethesda II thyroid nodules are referred to surgery due to their large size. Indeed, offering surgery for nodules ≥4 cm is a standard practice in several settings [15]. Although some reports have indicated a direct association between malignancy risk and nodule size [16,17,18], other studies have shown opposite results [15,19,20,21,22,23]. Thus, Cavallo et al. [20] and Magister et al. [21] observed an inverse relationship between nodule size and malignancy risk, with nodules <2 cm presenting the highest risk of malignancy, which was similar to our findings. Some authors, indeed, have advised against routine surgery referral for thyroid nodules ≥3–4 cm [15,21,24]. However, in a recent study, Hong et al. observed that nodule size impacts malignancy risk differently depending on the US pattern; whereas in nodules with a low or intermediate level of suspicion a size ≥3 cm increased the malignancy risk, this relationship was not observed in highly suspicious nodules [18].

Concerning sonographic characteristics, isoechoic but not hypoechoic nodules presented higher odds of being analyzed by FNA in our population, despite hypoechogenicity being related to the highest risk of malignancy [25]. This finding could be, again, due to the confounding factor of nodule size, as larger nodules (more commonly isoechoic) undergo FNA more frequently than subcentimetric nodules (more likely to be hypoechoic) [26]. In addition, hypoechogenicity is considered a less specific sign of malignancy than other sonographic characteristics (up to 55% of benign nodules are hypoechoic) [1].

On the other hand, the presence of microcalcifications is a well-known risk factor for malignancy in thyroid nodules, presenting an OR for malignancy of 6.7 in a large meta-analysis performed by Campanella et al. [25]. In addition, in our population, the finding of microcalcifications in a thyroid nodule indicated 11-fold increased odds of performing FNA, and we also observed that thyroid nodules with microcalcifications had a higher risk of being Bethesda IV-V-VI (OR 2.2). However, although malignant thyroid nodules presented significantly higher odds of microcalcifications in the bivariate analysis, multivariate regression analysis did not show any significant differences, probably due to the overall low prevalence of microcalcifications.

Regarding thyroid nodule composition, 82%–91% of thyroid cancers are solid [1], conferring a 4.7-fold increased risk of malignancy [25]. Accordingly, in our study, solid thyroid nodules had a two-fold increased risk of malignancy. Last, the finding of irregular margins has been recognized as a well-known risk factor for malignancy in thyroid nodules in some meta-analyses (OR, 6.12) [25]. Similarly, in our study, irregular margins were closely related to an increased risk of malignancy in the multivariate analysis.

High-resolution clinical units have been widely implemented in some areas of medicine [27,28], especially in the cardiology area, showing positive results, with 70% of patients obtaining a diagnosis and treatment of their medical condition on the same day [29]. Similarly, these high-resolution nodule clinics have been shown not only to be cost-effective [29] but also to reduce the delay in diagnosis and to improve patients’ satisfaction [9,29]. Along this line, our nondiagnostic FNA (Bethesda I) rate (4.3%) was lower than the usually reported rate of 8%–20% in several studies [30]. Previously, Díaz-Soto et al., in a single-center HR-TNC study, found a similar percentage of inadequate samples for cytology (4%) [29]. Possibly, positive outcomes of HR-TNCs could emerge from the participation of experienced and qualified professionals in the patient care process and by the characteristic design of HR-TNCs.

Our study has certain limitations but also some important strengths. A limitation of our study is the inherent nature of the study; due to the cross-sectional design, only associations, not causality, can be inferred. Another potential limitation is the multicenter design, which could imply different protocols and inhomogeneous practices between participating centers. In addition, we have not determined thyroid peroxidase (TPO) antibodies or collected information about head and neck radiation. On the other hand, the strengths of our study are the large sample size, the inclusion of data from HR-TNCs only, and the participation of highly trained professionals in thyroid nodule care.

In conclusion, in this multicenter study involving four HR-TNCs in Spain, we found that the presence of a solid component and irregular margins are independently associated with thyroid nodule malignancy. However, a nodule size ≥40 mm, despite being closely related to the indication of FNA, is associated with the lowest odds of malignancy. Therefore, a nodule size ≥40 mm should not be a factor leading to indicate surgery in thyroid nodules. Finally, we showed that HR-TNCs are associated with a low rate of nondiagnostic FNA results.

## Figures and Tables

**Table 1:** Clinical, ultrasound and cytopathological characteristics of thyroid nodules.

	n = 2809
Age (years)	51.9 ± 14.3
Sex (%)	
Male	17.3
Female	82.7
Nodule size (mm)	21.8 ± 12.3
Nodule size category (%)	
<20 mm	49.2
≥20–<40 mm	40.1
≥40 mm	10.7
Palpable (%)	
Yes	47.3
No	52.7
Echogenicity (%)	
Hyperechoic	8.1
Isoechoic	33.9
Hypoechoic	58
Composition (%)	
Mixed cystic and solid	29.6
Solid	70.4
Margins (%)	
Regular	96
Irregular	4
Microcalcifications (%)	
Absent	92.7
Present	7.3
Bethesda classification (%) (n = 2308)	
I	4.3
II	81.2
III	6.1
IV	5.3
V	0.9
VI	2.2

Data are the mean ± SD and the number (percentage) for categorical variables.

**Table 2:** Comparison between groups: FNA+ vs. FNA−, Bethesda II vs. Bethesda IV-V-VI, benign vs. malignant.

	FNA+	FNA−	*p*	Bethesda II	Bethesda IV-V-VI	*p*	Benign	Malignant	*p*
	n = 2308	n = 501		n = 1874	n = 194		n = 450	n = 135	
Age (years)	51.5 ± 14.1	53.4 ± 14.7	**0.011**	51.7 ± 13.8	50.1 ± 16.3	0.224	49.4 ± 13.4	46.8 ± 15.8	0.073
Sex (%)			0.719			0.122			0.637
Male	13.8	14.4		12	16.1		17	19	
Female	86.2	85.6		88	83.9		83	81	
Nodule size (mm)	24.2 ± 11.7	10.9 ± 7.9	**<0.001**	24.1 ± 11.7	25.6 ± 12.1	0.074	32.8 ± 12.1	24.4 ± 11.2	**<0.001**
Nodule size (mm)			**<0.001**			0.449			**<0.001**
<20	41	87.5		41.2	36.5		14.7	38	
≥20–<40	46.4	10.8		46.3	50		52.8	52.9	
≥40	12.6	1.6		12.6	13.5		32.5	9.1	
Palpable (%)			**<0.001**			**0.009**			0.055
Yes	56	17		55.8	68		73.1	62.2	
No	44	83		44.2	32		26.9	37.8	
Echogenicity (%)			**<0.001**			0.392			**0.012**
Hyperechoic	7.4	11.8		7.7	7		8.4	5.4	
Isoechoic	37.6	16.7		38.4	32		33.6	14.3	
Hypoechoic	55	71.5		53.9	61		58	80.4	
Composition (%)			**0.002**			**<0.001**			**<0.001**
Mixed cystic and solid	31.2	23.5		34.2	16.5		31	13.1	
Solid	68.8	76.5		65.8	83.5		69	86.9	
Margins (%)			0.250			**0.001**			**<0.001**
Regular	95.8	97.1		96.3	90.7		96.9	84.5	
Irregular	4.2	2.9		3.7	9.3		3.1	15.5	
Microcalcifications (%)			**<0.001**			**<0.001**			**<0.001**
Absent	91.7	98.4		93	81.6		90.2	76.1	
Present	8.3	1.6		7	18.4		9.8	23.9	

Data are the mean ± SD and the number (percentage) for categorical variables. FNA: fine-needle aspiration; OR: odds ratio.

**Table 3:** Bivariate and multivariate logistic regression analysis: odds for FNA.

	Bivariate			Multivariate		
Independent Variables	OR	95% CI	*p*	OR	95% CI	*p*
Age (years)	0.991	0.984–0.998	**0.009**	0.988	0.975–1.001	0.062
Sex			0.716			0.071
Male	1 (ref.)			1 (ref.)		
Female	1.053	0.797–1.393		1.534	0.964–2.440	
Nodule size (mm)	1.236	1.210–1.263	**<0.001**	-	-	-
Nodule size (mm)			**<0.001**			**<0.001**
<20	1 (ref.)			1 (ref.)		
≥20–<40	9.13	6.774–12.305	**<0.001**	10.97	6.108–19.702	**<0.001**
≥40	16.501	8.100–33.614	**<0.001**	34.476	4.734–251.073	**<0.001**
Echogenicity			**<0.001**			**0.002**
Hyperechoic	1 (ref.)			1 (ref.)		
Isoechoic	3.615	2.089–6.256	**<0.001**	2.385	1.233–4.614	**0.010**
Hypoechoic	1.234	0.772–1.973	0.38	1.14	0.643–2.021	0.654
Composition			**0.002**	-	-	-
Mixed cystic and solid	1(ref.)					
Solid	0.677	0.525–0.872				
Margins			0.252	-	-	-
Regular	1 (ref.)					
Irregular	1.452	0.767–2.751				
Microcalcifications			**<0.001**			**0.001**
Absent	1 (ref.)			1 (ref.)		
Present	5.505	2.418–12.532		10.931	2.607–45.829	

FNA: fine-needle aspiration; OR: odds ratio.

**Table 4:** Bivariate and multivariate logistic regression analysis: odds of Bethesda IV-V-VI.

	Bivariate			Multivariate		
Independent Variables	OR	95% CI	*p*	OR	95% CI	*p*
Age (years)	0.992	0.980–1.002	0.158	0.992	0.977–1.007	0.283
Sex			0.124			0.151
Male	1 (ref.)			1 (ref.)		
Female	0.714	0.465–1.096		0.64	0.348–1.177	
Nodule size (mm)	1.01	0.998–1.023	0.09	-	-	-
Nodule size (mm)			0.45	-	-	-
<20	1 (ref.)					
≥20–<40	1.22	0.883–1.685	0.227			
≥40	1.218	0.759–1.955	0.414			
Echogenicity			0.394	-	-	-
Hyperechoic	1 (ref.)					
Isoechoic	0.92	0.390–2.171	0.849			
Hypoechoic	1.25	0.549–2.847	0.568			
Composition			**<0.001**			**0.004**
Mixed cystic and solid	1 (ref.)			1 (ref.)		
Solid	2.625	1.621–4.251		2.217	1.297–3.791	
Margins			**0.001**			**0.021**
Regular	1 (ref.)			1 (ref.)		
Irregular	2.712	1.477–4.980		2.592	1.156–5.813	
Microcalcifications			**<0.001**			**0.006**
Absent	1 (ref.)			1 (ref.)		
Present	2.976	1.906–4.648		2.256	1.262–4.033	

OR: odds ratio.

**Table 5:** Bivariate and multivariate logistic regression analysis: odds of malignancy.

	Bivariate			Multivariate		
Independent Variables	OR	95% CI	*p*	OR	95% CI	*p*
Age (years)	0.987	0.972–1.001	0.073	0.991	0.971–1.011	0.359
Sex (%)			0.637			0.149
Male	1 (ref.)			1 (ref.)		
Female	0.872	0.494–1.539		0.578	0.275–1.216	
Nodule size (mm)	0.938	0.920–0.957	**<0.001**	-	-	-
Nodule size (mm)			**<0.001**			**0.001**
≥40	1 (ref.)			1 (ref)		
≥20–<40	3.579	1.831–6.995	**<0.001**	3.329	1.284–8.630	**0.013**
<20	9.26	4.526–18.944	**<0.001**	6.537	2.352–18.167	**<0.001**
Echogenicity (%)			**0.015**	-	-	**-**
Hyperechoic	1 (ref.)					
Isoechoic	0.667	0.153–2.899	0.589			
Hypoechoic	2.169	0.582–8.087	0.249			
Composition			**0.001**			**0.020**
Mixed cystic and solid	1 (ref.)			1 (ref.)		
Solid	2.974	1.595–5.546		2.313	1.138–4.701	
Margins (%)			**<0.001**			**0.003**
Regular	1 (ref.)			1 (ref.)		
Irregular	5.786	2.439–13.723		5.655	1.979–16.158	
Microcalcifications (%)			**0.001**	-	-	-
Absent	1 (ref.)					
Present	2.892	1.564–5.346				

OR: odds ratio.
